# Using Digital Technology to Reduce the Prevalence of Mental Health Disorders in Populations: Time for a New Approach

**DOI:** 10.2196/17493

**Published:** 2020-07-24

**Authors:** C Barr Taylor, Josef I Ruzek, Ellen E Fitzsimmons-Craft, Shiri Sadeh-Sharvit, Naira Topooco, Ruth Striegel Weissman, Daniel Eisenberg, David Mohr, Andrea Graham, Corinna Jacobi, Brian Oldenburg

**Affiliations:** 1 Stanford University Palo Alto University Stanford, CA United States; 2 Palo Alto University Palo Alto, CA United States; 3 Division of Hematology Oncology Department of Pediatrics Washington University St. Louis, MO United States; 4 Baruch Ivcher School of Psychology Herzliya Israel; 5 Linköping University Linköping Sweden; 6 Wesleyan University Middletown, CT United States; 7 Department of Psychiatry University of Michigan Ann Arbor, MI United States; 8 Northwestern University Chicago, IL United States; 9 Technical University of Dresden Dresden Germany; 10 The University of Melbourne Melbourne Australia

**Keywords:** internet, mental health, interventions, outcome, prevalence

## Abstract

Digital technology, which includes the collection, analysis, and use of data from a variety of digital devices, has the potential to reduce the prevalence of disorders and improve mental health in populations. Among the many advantages of digital technology is that it allows preventive and clinical interventions, both of which are needed to reduce the prevalence of mental health disorders, to be feasibly integrated into health care and community delivery systems and delivered at scale. However, the use of digital technology also presents several challenges, including how systems can manage and implement interventions in a rapidly changing digital environment and handle critical issues that affect population-wide outcomes, including reaching the targeted population, obtaining meaningful levels of uptake and use of interventions, and achieving significant outcomes. We describe a possible solution, which is to have an outcome optimization team that focuses on the dynamic use of data to adapt interventions for populations, while at the same time, addressing the complex relationships among reach, uptake, use, and outcome. We use the example of eating disorders in young people to illustrate how this solution could be implemented at scale. We also discuss system, practitioner-related, and other issues related to the adaptation of such an approach. Digital technology has great potential for facilitating the reduction of mental illness rates in populations. However, achieving this goal will require the implementation of new approaches. As a solution, we argue for the need to create outcome optimization teams, tasked with integrating data from various sources and using advanced data analytics and new designs to develop interventions/strategies to increase reach, uptake, use/engagement, and outcomes for both preventive and treatment interventions.

## Introduction

Digital technology, which includes the collection, analysis, and use of data from a variety of digital devices and other sources, has the potential to reduce the prevalence of mental health disorders and improve mental health in populations by integrating preventive interventions to reduce incidence with clinical interventions to reduce existing cases, both of which are needed to reduce the prevalence of mental health disorders, into health care and community delivery systems. Furthermore, digital technology has the advantage of collecting large amounts of information that can inform preventive and intervention processes. Such data need to be analyzed and, even more importantly, used to adapt and improve a variety of interventions, including digital ones, toward optimizing outcomes. The process of optimizing outcomes needs to be dynamic and responsive to the rapid changes in the use of digital technology, consumer interests and preferences, and regulations among other factors as well as to enable rapid changes in the content and process of intervention delivery (a study by Michie et al [1] provides an excellent overview of challenges in developing and evaluating digital interventions targeting behavior change as well as methods for doing so). At the same time, interventions need to be designed in partnership with stakeholders and consumers to increase the likelihood of subsequent implementation and dissemination [2-6].

The need to find novel solutions for improving mental health outcomes in populations is based on extensive data, showing that mental health disorders are very common and severely undertreated. In the United States, for instance, approximately 25% of the population experiences a mental health disorder during a given year and 50% in their lifetimes [7]; however, fewer than 30% of individuals with mental health disorders receive any treatment [8,9]. Thus, solutions for reducing prevalence in populations will require approaches that can be applied on a large scale [10].

To meet the need for outcome optimization at a population level, new approaches are required. Digital interventions have been developed to increase access and reduce costs, but engagement with these interventions is suboptimal, with many individuals engaging in only 1 or 2 sessions and fewer than half completing more than half of the treatment [11]. A recent study, for instance, of 93 mental health apps found that the medians of app 15-day and 30-day retention rates were 3.9% (IQR 10.3%) and 3.3% (IQR 6.2%), respectively [12]. To improve population-level outcomes, we have recently argued for the need for outcome optimization at the population level—an approach that simultaneously attempts to improve reach, uptake, engagement, and outcomes [1,13,14]. In this paper, we discuss how prevalence reduction in whole populations might be achieved through outcome optimization applied to both preventive and clinical interventions and suggest ways to address potential issues raised by this approach. Of note, outcome optimization applies to the entire population rather than the individual, and as we are focusing on prevalence, a reduction in caseness. However, as discussed later, the approach applies as well to any mental health or behavioral outcomes. We will argue that outcome optimization needs to be directed by a group of individuals with a diverse set of skills, which we refer to as an outcome optimization team, tasked with both reducing prevalence and incidence of a disorder in a population. Such teams would integrate data from various sources to increase reach, uptake, use/engagement, and outcomes for both preventive and clinical interventions and consider interactions/trade-offs among the variables. In a population model, focusing on incremental improvements in effect size may have much less impact on prevalence than focusing on increasing reach. For instance, Moessner and Bauer [15] noted that an increase of 10% from 10% in treatment utilization would decrease the number of cases by an additional 5%, whereas an improvement in treatment efficacy of 10% (from 10%) would only reduce the number of cases by approximately 2%. However, if the intervention used is not effective whatsoever or of very limited effectiveness, there would not be any population health benefits, irrespective of reach, uptake, and use, and there may also be harm associated with the intervention. Therefore, when considering how to optimize an intervention effect on a population, the aim should be to focus on reach while also considering how to improve efficacy.

## The Overall Approach

### Prevalence Reduction

The prevalence of a disorder in a population is measured by two primary factors: the number of individuals who are identified with the disorder and the number of individuals who develop the disorder during a defined period. To reduce prevalence, there are two necessary components: preventive interventions that reduce disorder onset (the incidence of new cases) and clinical interventions that are effective enough so that the individual already affected with the disorder no longer meets the clinical criteria for that condition.

A core premise of the approach we are suggesting is the recognition that interventions evaluated under carefully controlled circumstances in selected, often convenience populations may not be as feasible or effective when applied more broadly [5,16,17]. Therefore, to reduce the burden of mental health disorders in populations by delivering interventions at scale, interventions need to be carefully and systematically adapted to the needs, requirements, and interests of different defined populations in a dynamic way. In the following sections, we discuss the necessary components to achieve prevalence reduction through an outcome optimization model and use the example of eating disorders in young people to illustrate what we are proposing. The general model we follow is described in detail in a paper by Wilfley et al [18]. In this model, individuals in various populations are identified via an evidence-based screening [19] that sorts respondents into the following categories: possible anorexia, possible other eating disorder, risk of an eating disorder, and low risk of an eating disorder. The screening tool then provides appropriate prevention or treatment recommendations.

### Outcome Optimization Teams

**Figure 1:**
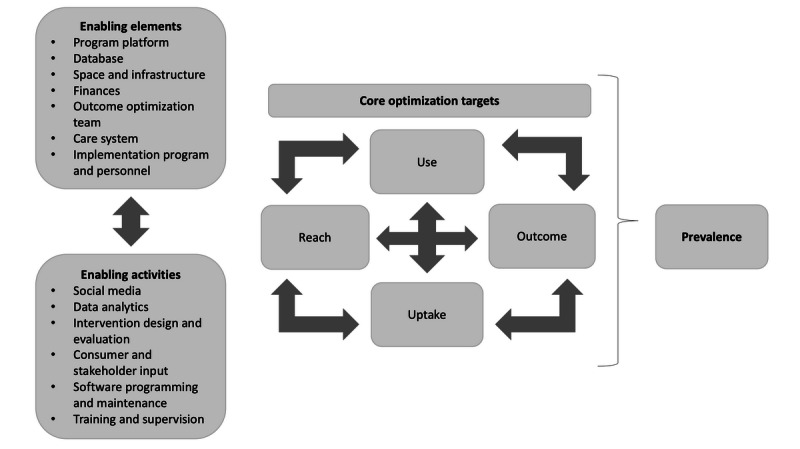
Outcome optimization model enabling elements, activities, and targets.

Affecting the key variables for optimizing outcomes is both a management and scientific process. As already mentioned, we have previously described the need for organizations to create *outcome optimization teams* or similar groups, tasked with monitoring the key variables mentioned in Figure 1 and developing and implementing strategies to improve the various targets and key outcomes. These teams must possess a set of specific skills to enable effective use of the data to improve the key outcomes, use novel trial designs to conduct rapid evaluations, and iteratively adapt available interventions to improve the effects of the interventions on all critical variables (ie, from reach to outcome). The outcome optimization team needs to include individuals who are able to address administrative issues, manage complex problems, design social media and intervention strategies, and analyze and interpret data, as well technology partners and oversight members (including consumers), brought together with adequate resources for the task. There are now many examples of applications of technology (eg, e-commerce) in areas not related to health and companies that now employ the equivalent of outcome optimization teams to one extent or another, with diverse skills and competencies, focusing on data science to help optimize the outcomes of interest. These approaches are equally relevant to population-level mental health interventions.

## Outcome Optimization Targets

In the optimization outcome approach, the primary components subject to intervention and evaluation (any of which could be targeted separately) are (1) *prevalence* of the disorder, the number of individuals in the population who are appropriate for a clinical intervention; (2) *risk* for the disorder, the number of individuals in the population who are appropriate for a preventive intervention (thus reducing incidence); (3) *reach*, the number of individuals in a population who are offered a relevant prevention or clinical intervention; and (4) *outcome*, the number of individuals who begin a program and achieve the desired outcome within a set time frame. The individual-level outcome would be categorical (case/no case), and the population-level outcome would be prevalence (defined as new cases and existing cases). In addition, two other variables are important for optimizing individual- and population-level outcomes: (5) *uptake*, how many of those individuals offered an intervention actually begin it (eg, create a user account for a web-based, guided self-help program and see a therapist); and (6) *engagement/use* (eg, how much of the intervention is used). To improve overall outcomes, we will argue that each of these parameters can be targeted individually and, yet, should be targeted and measured simultaneously, given that changes in one variable can have a downstream impact on another variable. For example, increased reach can reduce uptake rates, as less motivated individuals may be screened/included, or delivery of more effective treatments characterized by greater demands on client participation might be associated with reduced engagement.

We illustrate the different elements of our proposed approach by drawing from our research with eating disorders. Eating disorders are common and disabling problems with the highest mortality rates of any psychiatric illness. The risk factors for eating disorders are well known and have been shown to reduce incidence when addressed [20], and a variety of effective and cost-effective clinical interventions are available [21-23]. Furthermore, we have already developed and implemented an evidence-based screening on the web to categorize college students as being at risk for or with an eating disorder. Following completion of the web-based screening completion, we have provided appropriate and effective digital interventions or referrals to in-person intervention when appropriate at scale [24].

### Reach

In this paper, reach represents the percentage of the population who are at risk for or who have a clinical disorder who complete a web-based screening and who are offered a preventive or clinical intervention appropriate to their needs and interests. We focus on reach via a web-based screening, but individuals at risk for or with clinical disorders could also be identified in other ways, including clinical interviews, algorithms, and, of course, through self-identification.

In the example of eating disorders, the goal of a prevalence reduction program should be to reach most individuals at risk for or with eating disorders. The prevalence of eating disorders in female college students has been estimated to be approximately 13.5% [25]; the rates for those at risk of eating disorders are even higher. For instance, Lipson et al [26] found that approximately 17.29% (1163/6723) of women in their survey had high weight and shape concerns, and many others had binge eating and/or weight and shape concerns without meeting clinical criteria. Conservatively, at least 25% of college-aged women are at risk for but do not have eating disorders. Unfortunately, reach in most populations is often very low. For instance, in our recent study, which used screening in college and universities to recruit women to use an eating disorder intervention, 1.42% (4284/300,613) of undergraduate women completed the web-based screening [27]. Thus, increasing reach is a high priority. An outcome optimization team might use three strategies to increase reach in the population. Hypothetically, the initial focus might be to provide screening through customary channels (eg, student listservs and flyers). In this model, approximately 5.00% (50/1000) of those with eating disorders completed the screening. To increase reach, the next optimization strategy might be to add the use of targeted Facebook advertisements, followed by using both targeted Facebook and Google advertisements. In the example, we assumed that adding Facebook advertisements increased the reach to 7.50% (75/1000), and by adding Google advertisements (strategy 3), the reach increased to 10.00% (100/1000). We realize that these examples are hypothetical, but there is very sparse literature on the use of different digital techniques (eg, social marketing) for increasing reach in targeted populations.

Ten percent is still far below the goal of reaching most of the population, and targeted Facebook and Google advertisements can be expensive. Other, perhaps more effective and less expensive approaches could be used. For instance, web-based screening for an entire student population for a variety of problems, including eating disorders, could be routinely required, or a web-based screening sent directly to individual student emails could be offered. The Healthy Minds Network routinely reaches approximately 16% of students using the latter approach [28]. The outcome optimization team should continuously experiment with new approaches for increasing overall reach and reach for subpopulations of interest.

### Uptake

Once individuals have completed screening and have been offered feedback that directs them to a tailored intervention, the next critical variable is the uptake of the intervention, defined as having at least *begun* an intervention, such as engaging with at least some of a digital intervention or attending at least one individual or group therapy session. The uptake of interventions can be quite low. However, in our recent study of college women, 75.5% (690/914) of female students who were eligible for the clinical trial agreed to be randomized; of those randomized to the intervention, 79.2% (305/385) began it [27]. Data from another study that provides prevention and clinical interventions to students in public universities in the state of Missouri found that uptake rates of the digital programs offered for both the high-risk population and clinical population were about 44.2% (420/955) and 49.3% (187/379), respectively [24]. In another study, 26% (16/61) of students with possible anorexia nervosa who were recommended to seek treatment reported doing so at follow-up [29], and Lipson et al [26] found that 18.0% (345/1916) of students at risk of an eating disorder referred to an indicated preventive program began it. Overall, available data on the uptake of interventions have varied quite widely and may be impacted by such features as the population screened and the accessibility and perceived helpfulness of the interventions offered.

In our example, we assumed that for the clinical population, 50% of individuals provided with a referral recommendation will at least go on the web once, see a provider, or in some other way, engage in an intervention. A strategy that lends itself to digital technology is to provide a set of interventions that can be adapted to different needs and interests, with different resources to subpopulations within the larger targeted population. In our hypothetical example, the outcome optimization team found that the baseline uptake rate (number of individuals who were offered an intervention who began it) was 50%. The outcome optimization team explored and enacted a number of options to increase uptake. First, 25% of the sample was eligible but expressed little intention of acting on the recommendation and was provided a brief motivational intervention. Second, approximately 5% of the sample was found to favor Spanish as a primary language. A Spanish language version was authored and offered to interested individuals. A consumer survey found that approximately 25% of the reached population would choose in-person therapy but reported not being able to afford it. As a way to address this issue, the outcome optimization team created an option where therapists trained in evidence-based treatments for eating disorder (eg, cognitive behavioral therapy–guided self-help) would be reimbursed for treating individuals sponsored by a nonprofit organization, and this was offered, randomly, to half the individuals who might want to it. Another issue revealed by the outcome optimization team through consumer needs assessments was that many individuals with eating disorders live in areas with no available treatment expertise for eating disorders and/or preferred teletherapy to face-to-face therapy. The outcome optimization team, thus, developed a teletherapy eating disorder program based on previous work. To increase motivation in future users who met the characteristics identified through a moderator analysis, a postscreen feedback message was then designed to inform them that previous users with characteristics similar to theirs had achieved positive outcomes as a result of the program. Of course, the issues affecting uptake are not mutually exclusive, and variables such as cost, language, and expectation of success may have different impacts on different individuals depending on their needs and interests. All subpopulations are part of the larger population, and the needs and relative importance of subpopulations will continue to change, reflecting, for instance, changing demographics in the targeted population. Uptake will also change depending on consumer needs and interests, available resources, cost, and other factors.

One of the challenges of population-based interventions is that little is actually known about what users may want. A general strategy is to first consider the interests/resources of the users/stakeholders, followed by using user-centered design [30] approaches to build out interventions designed with ultimate implementation in mind [5]. In building out the interventions, modern designs would be used (eg, the multiphase optimization strategy and others [1,2,31,32] to engineer optimized interventions, before evaluation in randomized controlled trials). Depending on the size of the targeted population, other general approaches could be examined in subsamples. For instance, futility studies of stepped-care approaches, which might appeal to organizations as a way of reducing costs, could be examined [18,33].

### Use/Engagement

Program use, often defined as engagement, is the next key variable to consider. Use includes several factors, such as the amount of the prescribed intervention used (eg, sessions of therapy attended and web-based sessions opened); application of the skills outside of the program; and/or sufficient use to lead to reductions in clinical targets, clinical status, risk factor reduction, or other indicators of significant improvement [11]. For example, the results might indicate that individuals who complete a specific amount of intervention might show clinically significant improvements. This amount might refer to the number of sessions attended in face-to-face therapy or pages opened in web-based interventions. Important to determining sufficient engagement is to examine progression within an intervention both for symptom improvement and successful adaptation of behaviors, skills, and other targets considered necessary for a meaningful outcome. Digital technology and analytic methods offer the potential to passively assess a number of variables that might affect engagement and outcome (eg, activity, sleep, and search history). This offers advantages over other types of intervention in the availability of extensive data related to program use and, therefore, easier determination of use cutoffs.

Perhaps the most important use problem is that of early dropout. Early dropout has been defined in various ways, but it is defined, for the purpose of this discussion, as individuals who begin an intervention but complete an insufficient amount of a program to have a positive outcome (however, even this definition is problematic as many individuals seem to benefit from simply beginning an intervention or using it for only a short time) [1]. Recent meta-analyses of web-based studies report high rates of early dropout, partly depending on the amount of support offered [34]. Individuals may drop out of an intervention for a variety of reasons. A major goal of the outcome optimization team would be to understand why people are dropping out (particularly before they achieve a meaningful outcome) and to enact strategies to reduce dropout rates. Furthermore, the outcome optimization team would monitor use/dropout rates as they relate to clinical or preventive outcomes to help determine when a problem exists.

As with other components of the intervention, strategies to reduce early dropout and increase rates of sufficient engagement could be developed and tested. For instance, in a substudy of our recently completed trial [12], we examined the relationship between early and later session completion (n=47): individuals who completed at least three sessions were likely to complete at least 50% of the total program. In another analysis, we found that a very high score on a measure of thin body ideal (TBI) internalization predicted students likely to drop out of the intervention early. We then examined the components of the first three sessions to see what might have negatively impacted 21 individuals with a high TBI. One component tasked users to write a letter to their body. Of these 21 individuals, 10 rated it as not useful at all, 10 as somewhat useful, and only one as very useful. Therefore, we dropped this technique from the program and added a motivational interviewing piece. A subsequent sample of the first 59 users of the motivational interviewing piece was reviewed. The component was rated as not helpful by 10% (6/10) of individuals, a bit helpful by 44% (26/60), helpful by 42% (25/60), and very helpful by 5% (3/60). A number of other small changes were made over the course of a year, and we found that completion rates for the first two sessions increased from 69.5% (105/131) to 78% (70/89) following implementation of all the changes.

Within a dynamic, monitored population, there are innumerable approaches to improving engagement, and methods to do so have been well described [1,5]. However, it is worth noting that as reach is successful in subpopulations, each of these subpopulations is likely to generate new issues of uptake, engagement, and outcome.

### Outcome

As with engagement, much has been written about how to improve outcomes using many of the strategies already discussed. The outcome optimization being discussed aligns with movements toward outcome-based care and measurement-based care [34], in which the delivery of interventions provided to clinical populations should focus on the use of outcomes to guide clinical decision making. We briefly mentioned what constitutes a positive outcome (eg, a clinically significant change) measured by loss of clinical status or even abstinence from eating disorder behavior. However, from a population-based prevalence reduction standpoint, there are some important considerations, as illustrated by the eating disorder program we are discussing. First, most of the studies on new models to improve outcome focus on digital interventions. Given some individuals’ preference for face-to-face interventions, other treatment modalities and methods should be considered—we mentioned teletherapy above, for instance, as one obvious example. Second, as mentioned previously, focusing on incremental improvements in effect size may have less impact on prevalence than focusing on increasing reach. Third, interventions should be selected and designed with implementation in mind [5]. Mohr et al [33] noted that when digital interventions that are shown to be efficacious in laboratory-based studies move to real-world settings, individuals do not engage with the tools and implementation often fails. Fourth, the complex issue of the ordering of interventions needs to be considered in population-based models. We, and others, have proposed stepped-care models as a *cost-effective* approach and also as a way to identify subpopulations that may need alternative approaches [14]. Thus, randomized controlled trials remain an important component of digital interventions. At the same time, our central thesis is that researchers must consider issues related to dissemination and implementation from the beginning [5]. This will lead to interventions that maximize public health impact.

## Refining the Public Health Model

The model overlaps with other public health models, such as the highly influential Reach, Effectiveness, Adoption, Implementation and Maintenance model [35]. Similar to other public health models [36], we stressed the importance of offering both prevention and intervention approaches to reduce the prevalence. The model proposed here differs from existing models in terms of focusing on the use of digital databases and digital health interventions and innovations in reducing the prevalence of targeted populations. These data and analytics need to be monitored and interpreted by a multidisciplinary team involved in program codesign and delivery so that the digital health solution and its delivery can be quickly refined and improved, an approach consistent with Mohr et al’s [5,17] recommendation that program evaluation should be more rapid and agile with iterative improvements. In the model we propose, the key innovation is that the key outcomes (reach, uptake, engagement, and outcome) are being simultaneously and continuously monitored, with ongoing testing and refining of strategies to improve these various indices. Fortunately, some emerging studies deal with at least several components of our proposed model. For instance, the Quit Sequential Multiple Assignment Randomization Trial Utah trial uses a cluster-randomized, multilevel sequential multiple assignment randomized model to examine strategies to increase reach and efficacy in community health centers [37].

## System-Level Issues

Our proposed approach has focused on identifying the key elements for reducing symptoms in individuals, to reduce the prevalence of eating disorders in a population. However, program delivery and access and uptake of interventions are affected by a variety of system-level issues, including regulations/policies (eg, reimbursement), service delivery (eg, availability of trained providers), social environment (eg, support of family/friends), infrastructure (eg, availability of exercise equipment for those who may want to use it), accessibility (eg, availability of reliable transportation), and, of course, funding and reimbursement. For example, building out a teletherapy option may be limited by professional practice guidelines and issues of practicing across state lines, reimbursement, access to privacy-protected systems, and/or professional attitudes and training. The outcome optimization team needs to take these factors into consideration so that possible strategies are considered in the context of what can be achieved within the system responsible for prevalence reduction. Another issue is that there may be divergent agendas across different levels of the organization. Providers, supervisors, division leaders, and organizational leadership may have different ideas about what a service should be doing or offering.

Another major challenge for the outcome optimization model is for the outcome optimization team to have access to platforms in which interventions can be easily offered and adapted for use in various formats (eg, mobile apps and web browser access). Furthermore, the delivery system needs to be integrated with a broader health care delivery system. An example of a system designed to integrate digital practice into a common health care record is now being implemented in Australia [38]. Such a system allows feedback between outcome optimization teams and health care practitioners.

## Putting It All Together: Prevalence Reduction Through Outcome Optimization

Using this approach at the population level has the potential not only to reduce population prevalence but also to provide important information about intervention and prevention mechanisms, subpopulation needs, and even how to provide personalized and customized interventions to individuals in a population. The model is based on data and data analyses, but most importantly, on having an expert and technically proficient team that is tasked with using data and novel trial designs to conduct ongoing redesign, implementation, and rapid evaluation of evolving interventions that adapt/provide for individuals’ needs and address the effects of interventions on all critical variables (ie, from reach to outcome) that can potentially result in greater overall reductions in prevalence. *The creation of outcome optimization teams within organizations tasked with reducing prevalence would be the single most important step toward optimizing outcomes.*

Our approach also assumes that there is agreed-upon access to users’ data and their willingness to provide information about their progress. The use of such data will need to be transparent, and the user will need to agree. Thorough and repeated efforts will need to be made to ensure that such information follows Health Insurance Portability and Accountability Act guidelines and remains confidential. The concerns of individuals about sharing data need to be taken seriously, and it is likely that many will opt out of data sharing, given the many examples of how personal data are being misused. However, there is also an increasing movement—sometimes referred to as the *quantified self* movement—toward health consumers having access to their own data and making this information available to their health providers and others to access.

## Limitations of Dichotomous Measures as Outcomes

Before discussing how an outcome optimization team model might be enacted, it is important to note that prevalence rates determined by caseness have a number of limitations. Psychopathology exists on a continuum, and individuals, over time, may fall in and out of risk and/or caseness. Continuous measures are much more likely to be informative than categorical measures. However, for the foreseeable future, prevalence reduction will be measured in terms of reduction/prevention of caseness; thus, the reduction of prevalence remains the most important goal of a population-based approach.

More broadly, digital technologies will need to and are able to address a number of other important issues besides any specific psychopathology or risk that is being addressed. First, dissemination and implementation research have tended to focus on single disorders. However, nearly all mental health problems presenting in practice have substantial comorbidities. In theory, one could reduce the prevalence of one disorder in an individual while having little effect on several other important problems. A number of studies are examining transdiagnostic approaches and considering outcomes across multiple categories and dimensions [12]. Second, being a case may be less important to the individual identified as such than other issues, such as quality of life and well-being. The inclusion of variables relevant to the individual should be part of an intervention system. Third, health behaviors and risk factors (eg, obesity) are intrinsically related to many disorders and should also be addressed. Unfortunately, few models of multicomponent, multidimensional interventions assessed over time have been reported. Fourth, individual-level data obtained through ecological momentary assessment and other techniques can be used as part of a population reduction strategy, and an outcome optimization team will need to consider both general and personalized interventions as part of an array of opportunities provided to individuals at risk for or with clinical disorders. The model we describe is applicable to multidimensional personalized interventions once models for doing so have been developed.

## A Note on Prevention

A major strength of digital technology and the use of an outcome optimization team is that screenings and other methods can be used to identify both individuals at risk for and/or with clinical disorders [39] and to address issues of reach, uptake, use, and outcome in at-risk populations using the methods discussed earlier. In most settings, we know of where screenings are used that could identify both risk and caseness; only the latter group is addressed. Not providing preventive interventions is a lost opportunity. We realize that there are many issues with doing so: the burden of adding prevention onto the responsibilities of clinical services, the challenge of providing prevention at a low cost given the large number of individuals who might need to be provided with interventions, and issues of reimbursement, to name a few. However, scalable interventions shown to reduce onset for common problems such as depression [39] and eating disorders [20] are available. Implementing, altering, and adapting them to optimize reach, uptake, use, and outcome (reduction in risk) using the methods we have described may be one of the most important challenges we face.

## How Reduction of Population-Wide Prevalence Could Happen

Efforts to reduce the population-wide prevalence of disorders will require considerable resources, and any effort to achieve such reductions will need to be aligned with government/institutional/provider/community perspectives and/or other values and goals. There are many health care systems (eg, Kaiser Permanente, the United States Department of Veteran Affairs) that consider prevalence reduction of disorders (eg, suicide, posttraumatic stress disorder [PTSD], and depression) within their populations to be of importance and routinely screen their populations for problems such as depression and PTSD. Reducing prevalence across populations is similarly critical to many governments and other institutions with missions to serve specific groups. For example, colleges and universities have a strong interest in reducing the prevalence of mental health disorders and ultimately reducing the number of individuals who drop out from college with the result that many could create outcome optimization teams focused on reducing these outcomes in their populations. Another option in health care systems is that groups of collaborative care teams could take responsibility for the outcome optimization of preventive and clinical interventions in their panels, sharing findings among the teams. However, deploying digital technologies focused on reach and uptake may create a major problem for the provider systems: it is likely to create a major increase in demand for services. As we go forward, considerations of the cost-effectiveness and creative design of service delivery models, including stepped-care approaches, will need to be layered into the models.

A population-wide prevalence reduction program also has implications for the health care system, where cost/benefits and trade-offs of combined prevention and intervention need to be considered and may complete competing interests, where, for instance, might be more interested in the short-term benefits of treating cases rather than preventing new cases and considering needs to be given to who directs the outcome optimization team goals.

An alternative approach to a total population-based approach is to focus first on outcome optimization within defined segments of the total population, for instance, individuals identified through screening. This approach does not focus on reductions in overall prevalence but only the reduction of risk and caseness within a subset of the entire population of interest. Working with the National Eating Disorders Association, we created a virtual outcome optimization team (comprising information scientists, statisticians, data managers, psychologists, psychiatrists, program designers, software providers, and others) to focus on both prevention and intervention for eating disorders for individuals identified through the National Eating Disorders Association screening tool. As an example of the need to enact new models of care delivery, over a 6-month period, 71,362 individuals completed the screening and most individuals (61,585/71,362, 86.30%) screened positive for an eating disorder. In addition, 10.20% (6602/71,362) were screened as being at high risk for the development of an eating disorder. Of those who screened positive for an eating disorder, 85.90% (52,902/61,585) had never received treatment and only 3.00% (1847/61,585) were currently in treatment [40].

As another step forward, funding agencies should actively support innovative population-based interventions that use *newer* designs, including just-in-time adaptive interventions and other ways of thinking about and using digital technology and data to improve outcomes. As noted previously, we remain advocates of traditional randomized controlled trials, when implemented following intervention optimization, and we see them as being most meaningful when instituted within populations where they might eventually be deployed. Funding agencies might initially focus not on a comprehensive population prevalence reduction model but initially examine issues that would be relevant to the model. The investigators would be required to focus on defined, large populations, reach, uptake, engagement, and outcomes using modern data analytics and methods—and many such studies are now underway—with a demonstration as to how, where, and when they would be implemented in real-world situations.

Implementing prevalence reduction in populations is a challenge, but given the large number of people in any population at risk for or with a clinical disorder, scalable, innovative models of service delivery are urgently required [16]. Digital technologies can enable scalability, but new systems and models need to be developed to take advantage of this capability. We have argued that the implementation of outcome optimization teams represents an important possible approach to enable the delivery of technology-facilitated mental health interventions in a way that can optimize outcomes for the entire population.

## Abbreviations

PTSD: posttraumatic stress disorder
TBI: thin body ideal
